# Supplementary material for: Perinatal Outcomes and Level of Labour Difficulty in Deliveries with Right and Left Foetal Position—A Preliminary Study
Source: Healthcare (Basel). 2024 Apr 22;12(8):864. doi: 10.3390/healthcare12080864 (PMC11049945; doi:10.3390/healthcare12080864)
Supplement: Supplementary file 1 [file healthcare-12-00864-s001.zip › study tool 1.pdf]

Labour Observation form

Start of observation:				Initials:		ID	
age:		pregnancy:		labour:		week:	
height:		Weight before pregnancy:		Weight before labour:			

**induction:**	YES	NO	**Indication:**	

**On the admission:**		
**USG**		**Leopold's manoeuvres:**
**Foetal positioning**	☐ right ☐ left ☐ unable to determine	☐ right
		☐ left
**Placenta location**	☐ anterior ☐ posteri ☐ funda ☐ right ☐ left	☐ unable to determine

**Assessment in the first stage of labour (every 2h or more often when necessary):**													
Time													
Uterine contractions/ 10 min													
regular/discoordination (R,D)													
Assessment of the cervix	Cervix consistency Firm (F) Medium (M) Soft (S) and dilation												
Engagement of foetal head	Position of cranial sutures and fontanelle (using clock)												
Progression of foetal head in relations to the interspinal line	graph												
epidural (+/-) + works/ - does not work													

**Assessment in the second stage of labour (every 15min or more often when necessary):**												
Time												
Engagement of foetal head	Position of cranial sutures and fontanelle (using clock)											

Labour Observation form

Progression of foetal head in relations to the interspinal line												
epidural (+/-)												

**Pelvis assessment:**	
	☐ **normal** ☐ **abnormal**

**Assessment of uterine contractions**: (more than one answer possible)

☐ PROM (no uterine contractions)

☐ primary hypotonic uterine contractions

☐ secondary uterine inertia

☐ primary hypertonic uterine contractions

☐ discoordination of uterine contractions

☐ contractions of normal streght, but shorter [illegible]

☐ normal uterine contractions

☐ normal uterine contractions after oxytocine

☐ normal uterine contractions after amiotomy

☐ normal uterine contractions after spontaneous rupture of the membranes

**How long was the uterine contractions (how many hours)**							
**irregular**		**every 10 minutes**		**every 5 minutes**		**every 2-3 minutes or more frequent**	

**Phases of labour (in minutes)**			
Latent phase of the first stage (< 4cm)		First stage (from contractions every 5 min)	
Second stage		Active pushing in the second stage	

☐ Lack of progress in the first stage of labour (dilation)…………. hours without progress……………………..

☐ Very slow progress

Lack of progress in the second stage of labour (more than 1 hours)

☐ yes, progression of the foetal head ……………………..

☐ no

When the woman experiences the urge to push?

☐ no urge to push

☐ in the first stage of labour (dilation?) …………………………………………

☐ in the second stage of labour (after how many minutes?) …………………………………………

**If epidural was used, what were the indications?**

Labour Observation form

☐ woman's request as initialy said in the birth plan		
Woman’s request because:<br>☐ pain too intense ☐ woman not feeling well psychologically<br>☐ lack of progress ☐ suggested by the personnel		
Does epidural changes the engagement and the rotation of foetal head?	YES	NO
Where the pain was mostly located	☐ sacrum ☐ back ☐ thighs ☐ lower abdomen<br>☐ other...	

**Delivery:**	☐ vaginal ☐ vacuum<br>☐ forceps ☐ CS
**Indications for instrumental or surgical delivery:**	☐ abnormal foetal heart pattern ☐ lack of labour progress<br>☐ prolonged second stage of labour ☐ woman's exhaustion<br>☐ lack of cooperation between woman and medical personnel<br>☐ lack of personnel's patience<br>☐ other...

**Birth:**							
Sex		weight		Apgar score		ICU	
Does the external foetal head rotation was spontaneous?				YES			
				NO			
Assessment of perineal injury :				☐ First-degree tear ☐ Second-degree tear ☐ Third-degree tear<br>☐ laceration of the vaginal wall<br>☐ laceration of the vaginal mucosa<br>☐ laceration of the periurethral labia<br>☐ minor abrasion<br>☐ other			
Assessment of perineal injury by a midwife:				☐ small ☐ avarage ☐ big			
**Blood loss**				☐ < 350 ml ☑ 400-500 ml ☐ >500 ml			
**Woman’s attitude before labour**				☐ very positive ☐ positive ☐ neutral ☐ negative ☐ very negative			
