# Supplementary material for: Perinatal Outcomes and Level of Labour Difficulty in Deliveries with Right and Left Foetal Position—A Preliminary Study
Source: Healthcare (Basel). 2024 Apr 22;12(8):864. doi: 10.3390/healthcare12080864 (PMC11049945; doi:10.3390/healthcare12080864)
Supplement: Supplementary file 1 [file healthcare-12-00864-s001.zip › study tool 2.pdf]

Assessment of the level of labour difficulty form

**How do you assess the level of labour difficulty on the scale from 1 to 10, where 1 refers to minimal and 10 to maximal difficulty?**	Score
**Assessment by a woman (1-10)**	
**Assessment by a midwife (1-10)**	
